# Factors Influencing the Participation of Shared Decision Making in Stable Coronary Artery Disease Patient: Protocol of a Mixed Methods Study

**DOI:** 10.3390/healthcare12181883

**Published:** 2024-09-20

**Authors:** Xiangxu Meng, Chengang Hong, Xingwei Zhang

**Affiliations:** School of Nursing, Hangzhou Normal University, Hangzhou 311121, China; mengxiangxu33@gmail.com (X.M.); hongchengang2022@163.com (C.H.)

**Keywords:** shared decision making, stable coronary artery disease, participation, the theory of planned behavior

## Abstract

Introduction: The “paternalistic decision-making model” is no longer well suited to the modern clinical environment, and therefore, shared decision making (SDM) has emerged as a key approach. Although the benefits of SDM have been largely reported, several studies have reported low participation in SDM in coronary artery disease (CAD) patients. The theory of planned behavior (TPB) model is one of the most frequently employed theoretical frameworks for predicting human behaviors. According to the TPB, intention is influenced by attitude, subjective norm, and perceived behavioral control, while behavior is influenced by both intention and perceived behavioral control. Therefore, we propose a mixed methods study based on TPB to investigate the status of Chinese stable coronary artery disease (SCAD) patients’ participation in SDM, understand their experiences of SDM, and explore the factors that influence their participation in SDM. Materials and Methods: An explanatory sequential mixed methods design will be used to explore the study aims, including a quantitative phase, a subsequent qualitative phase, and the final integration study. The quantitative study will use convenient sampling from the Affiliated Hospital of Hangzhou Normal University to conduct a cross-sectional survey (n ≥ 252). The qualitative study will be sampled using the maximum difference sampling method from the quantitative study results, and then the data will be collected through semi-structured interviews (n = 10–20). This study will use descriptive statistics and test hypotheses while considering a *p*-value of <0.05, which will be considered statistically significant. Discussions: The study employs a mixed method approach with an explanatory sequential design, incorporating qualitative and quantitative methods to comprehensively understand the factors influencing SCAD patients’ participation in SDM. Furthermore, these findings can inform the design of future intervention studies and provide healthcare providers with targeted information and communication to help SCAD patients make the most appropriate decisions. Study participants will be recruited using convenience sampling from just one single clinical setting, which may limit the findings’ generalizability. Ethics and Dissemination: This study has been approved by the Ethical Committee of the School of Nursing, Hangzhou Normal University (Approval No.: 2024013). All the participants will sign an informed consent form before participating in the survey. The corresponding results and conclusions will be disseminated in journals and conferences after the completion of the study.

## 1. Introduction

Paternalistic decision making can be defined as a situation in which a physician does not fully comprehend the patient’s needs, interests, and preferences [1]. In such instances, the physician may attempt to influence the patient’s actions and decisions on the grounds that they believe it is in the patient’s best interest to do so. In recent decades, advances in medical technology and the updating of medical devices have led to more options for the diagnosis and treatment of diseases, while patients’ access to medical information has become more diverse, and the awareness of their rights has increased significantly. These have led to major changes in medical decision making. The “paternalistic decision-making model”, which has long been the dominant paradigm in medical practice, is no longer well suited to the modern clinical environment, and therefore, shared decision making (SDM) has emerged as a key approach [2]. SDM facilitates a thorough comprehension of treatment choices and associated risks by involving healthcare professionals and patients, emphasizing the importance of disseminating the best available evidence and acknowledging individuals’ and their families’ needs, values, and experiences when making decisions [3,4]. SDM serves as an intermediary approach, bridging the gap between a completely paternalistic model and an entirely patient-driven one. This method fosters an equitable partnership where both the healthcare provider and the patient jointly assume responsibility for making medical decisions [5].

Cardiovascular disease (CVD) represents a significant contributor to mortality, with deaths attributable to CVD representing an increasingly critical concern for global health [6]. As the Global Burden of Disease study indicated, in 2022 alone, CVD caused an estimated 19.8 million deaths worldwide [7]. In China, the number of people suffering from cardiovascular diseases has reached 330 million, and the mortality rate accounts for about 40% of the national mortality rate, of which coronary artery disease (CAD) accounts for 11.39 million, which is the main cause of the death and disability of Chinese residents [8]. Stable coronary artery disease (SCAD) is the most common type of CAD.

Patients with SCAD frequently present with symptoms that impair their quality of life. Common treatment options include optimal medication therapy (OMT) and coronary revascularization therapy [9]. It has been demonstrated that the range of treatment options for SCAD is, in the majority of cases, comparable [10]. In other words, there was no significant difference between the two in terms of long-term symptom relief or reduced risk of heart attack or death. Furthermore, the decision regarding the most appropriate course of treatment is often a matter of personal preference, with different patients requiring and desiring different forms of treatment. SDM not only improves treatment adherence and health outcomes but also directly impacts the patient experience, healthcare satisfaction, and quality of life (QoL) [11]. Multiple cardiovascular guidelines recommend shared decision making for SCAD patients who are capable of autonomous decision making [12,13,14]. Although the benefits of SDM have been largely reported [15], several studies have reported low levels of participation in SDM in CAD patients [12,16,17]. One study revealed that merely 10% of the patients undergoing percutaneous coronary intervention (PCI) are informed of alternative options, only 19% are apprised of potential drawbacks, and just 16% are consulted about their treatment preferences [12]. In the Chinese context, there is a lack of evidence on the factors influencing the participation of SCAD patients in SDM.

The planned behavior theory (TPB) model is often used as a theoretical framework to predict human behavior. The theory posits that observing human behaviors involves a rational decision-making process and asserts that intention dictates behavior. Attitude, perceived behavioral control, and subjective norms have been documented as positively influencing behavior via intention [17]. TPB has been widely used to explain the behavioral intentions and behaviors of various diseases and populations, including physical activity [18], smoking behavior [19], dietary behaviors [20], contraceptive decisions [21], and breastfeeding decision making [22]. However, the factors that comprise the TPB domains among SCAD patients’ participation in SDM are unclear.

Therefore, we propose a mixed methods study based on TPB to investigate the current status of Chinese SCAD patients’ participation in SDM, understand their experiences of SDM, and explore the factors that influence their participation in SDM. Our greater goal is to provide important information and references for designing specific interventions and scaling up SDM in the future.

## 2. Materials and Methods

### 2.1. Research Questions

The objectives of the study are as follows: What is the current status of Chinese SCAD patient’s participation in SDM?

What are the experiences of Chinese SCAD patient’s participation in SDM?

What are the factors influencing Chinese SCAD patients’ participation in SDM?

### 2.2. Theoretical Framework

**Figure 1:**
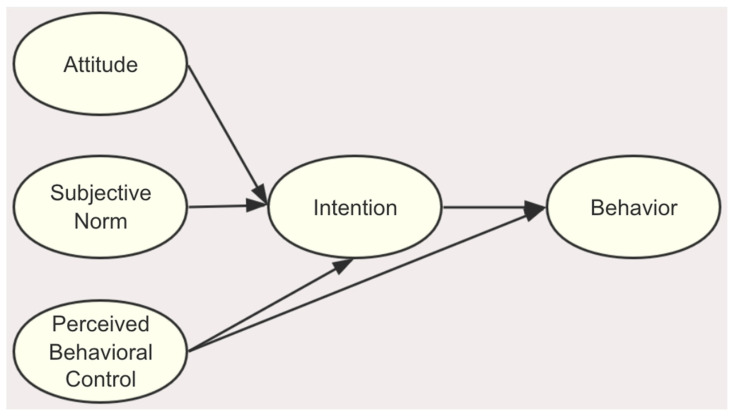
The theory of planned behavior.

The theory of planned behavior (TBP) framework was adopted in this study (Figure 1). According to the TPB, intention is influenced by attitude, subjective norm, and perceived behavioral control, while behavior is influenced by both intention and perceived behavioral control [23]. Attitude is a comprehensive assessment of performing a certain behavior. In this study are views on the benefits, disadvantages, and outcomes of participating in SDM behavior.

Subjective norm refers to the social pressure exerted by significant others for someone to engage in a particular behavior and their level of support for that behavior. In this study, it was defined as the degree to which SCAD patients felt the pressure of their significant other and the support they provided when deciding whether to engage in SDM behavior.

Perceived behavioral control measures how a person perceives the ease or difficulty in performing a behavior. Perceived behavioral control increases with individuals’ perceptions of having greater resources and opportunities available to them, along with fewer perceived obstacles. In this study, it is defined as the resources, opportunities, and obstacles that SCAD patients perceive when they participate in SDM behavior.

### 2.3. Design

**Figure 2:**
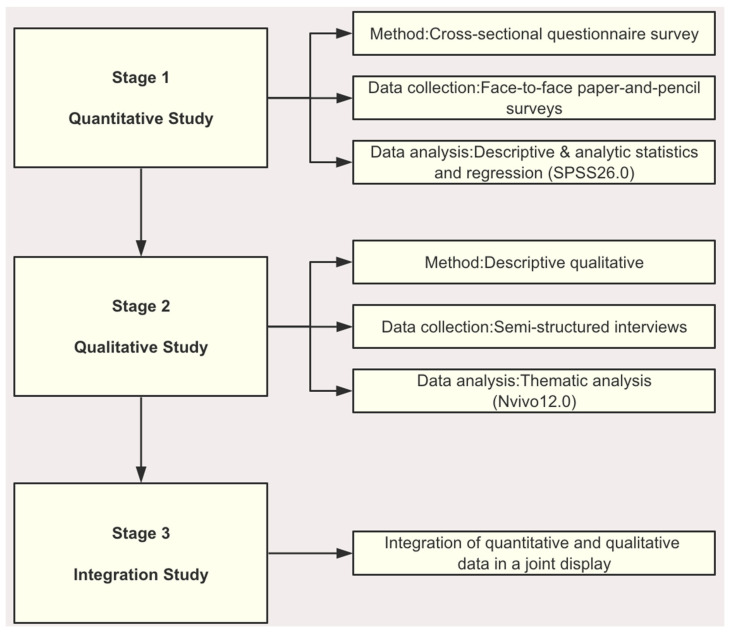
Flow chart of the mixed method study.

An explanatory sequential mixed methods design will be used to explore the study aims, including a quantitative phase, a subsequent qualitative phase, and the final integration study (Figure 2). In the quantitative phase of the study, quantitative data on the status of participation in SDM among SCAD patients and the factors influencing it will be collected in large quantities through a cross-sectional study. In the qualitative phase of the study, semi-structured interviews with SCAD patients representing the quantitative results will be conducted through a descriptive qualitative study to deeply explore their subjective feelings and experiences of participating in SDM. Thematic analysis will be used to explore the factors influencing their participation in SDM, which further supplemented the quantitative study. Finally, the results collected in the quantitative and qualitative phases will be integrated, and the statistics-by-themes and side-by-side comparisons will be used in a joint display.

### 2.4. Stage 1: Quantitative Study

#### 2.4.1. Sample

A convenience sampling method will be used to recruit participants. The study included 14 demographic and clinical characteristics and seven dimensions corresponding to five formative scales, with a total of 21 variables (Table 1). According to the sample estimation algorithm proposed by Kendall [24], the sample size is 10–20 times the number of variables, and the minimum sample size of this quantitative survey is 210. Moreover, considering 20% of the lost follow-up rate and invalid questionnaires, the final minimum sample size for the study is 252.

The inclusion criteria are as follows: (1) meets the diagnostic criteria of SCAD in the Guidelines for Diagnosis and Treatment of Stable Coronary Heart Disease [25]; (2) ≥18 years old; (3) signed informed consent.

The exclusion criteria are as follows: (1) patients with emergency admissions; (2) patients fully delegated to the family decision-maker; (3) patients with communication disorders; (4) diagnosed with mental illnesses; (5) pregnant patients; (6) patients with severe cardiac, liver, or renal dysfunction or critical illness.

#### 2.4.2. Study Setting and Data Collection

The study will be conducted at the Affiliated Hospital of Hangzhou Normal University in Hangzhou, Zhejiang Province, China.

Before the questionnaire survey, team members will receive unified training to master the purpose, method, content, and process of the questionnaire survey. When answering questions from patients, unified guidance language should be used to ensure the authenticity of answers and the reliability of data. With the support and cooperation of hospital staff and the consent of SCAD patients, investigators will require the participants to sign informed consent forms and provide their telephone numbers through which they can be contacted for the next stage. We will be preparing small gifts for patients to stimulate their enthusiasm to participate in the survey.

Data collection stage: One or two days before discharge, the researchers and participants will conduct face-to-face paper-and-pencil surveys to fill out questionnaires. The questionnaire survey will take about 10–15 min. For elderly patients with low educational levels, the researchers will clearly explain the contents of each survey and then ask the offspring of the patients to fill in the questionnaires. The questionnaires will be issued on the spot, and the questionnaires will be collected on the spot and checked in time to ensure the completeness and effectiveness of data collection.

#### 2.4.3. Instruments

Data collection will be performed by using the following instruments:

(1) General information questionnaire: Demographic and clinical characteristic data will be obtained by the general information questionnaire. Demographic characteristics will include age, sex, BMI, ethnicity, marital status, education level, average annual household income, place of residence, working conditions, medical payment method, drinking status, and smoking status. Clinical characteristics will include the duration of illness, the first time to be hospitalized for SCAD or not, the severity of angina, stent implantation or not, and the number of diseased coronary arteries.
(2) Questionnaire for measuring patient views of involvement in myocardial infarction care [26]: This questionnaire was developed by Judith E, and translated by H. Shen [27]. Patient attitudes towards SDM will be measured by the patient involvement section of this scale, which consists of 6 items. The responses to each item are scored from 1 (Don’t agree at all) to 4 (Agree completely) with the total scores ranging from 6 to 24 points. A higher score indicates a more positive attitude towards SDM on the part of the patient. Cronbach’s α for the subscale is 0.82, indicating good internal consistency reliability.
(3) Decision Self-Efficacy Scale (DSES) [28]: DSES was developed by Bunn et al., and translated by S.T. Wang et al. [29]. Decision self-efficacy is the “self-confidence or belief in one’s abilities in SDM”, and we will use DSES to assess the self-efficacy of participants towards SDM. The response options will be measured on the 5-point Likert scale (0 = not at all confident to 4 = very confident). On the advice of the original author, the total self-efficacy score is obtained by summing the 11 items and then multiplying them by 11/25. The total score ranges from 0 (not confident) to 100 (extremely confident). DESE demonstrated excellent reliability in the original study (Cronbach’s α = 0.899). The English version of DSES was translated into Chinese, back-translated, and cross-culturally adapted according to Brisin’s principles. The Chinese version of DSES consisted of 11 items. The content validity index of the Chinese version scale was 0.966. The Cronbach’s α coefficient was 0.918. The correlation coefficients between the scores of each item and the total score were 0.627–0.796 (all *p* < 0.01).
(4) The Autonomy Preference Index (API) [30]: API was developed by Ende, and translated by Kim et al. [31]. The intention was assessed by 6 items using responses on the 5-point Likert scale (1 = “strongly disagree” to 5 = “strongly agree”). Because scoring for these items is reversed, a lower score indicates a greater intention. The internal consistency reliability of the subscale was excellent (Cronbach’s α = 0.82), and the test–retest reliability was 0.84.
(5) The 9-item Shared Decision-Making Questionnaire (SDM-Q-9) [32]: SDM-Q-9 was developed by Kriston, and translated by B.H. Luo et al. [33]. The English version of SDM-Q-9 was translated into Chinese, back-translated, and cross-culturally adapted according to Brisin’s principles. The degree of the patient’s actual participation in SDM during treatment will be assessed by 9 items using answers on the 6-point Likert questionnaire ranging from 1 to 6 (“completely disagree” to “completely agree”). The SDM-Q-9 showed excellent validity and high acceptance, and internal consistency yielded a Cronbach’s α of 0.938 in the test sample. The initial author suggested that the original score × 20/9 be converted to 0–100 points for easy comparison. The total scores of 0–33, 34–66, and 67 and above indicated low, medium, and high levels of patient participation in shared decision making, respectively. Higher scores indicate a greater degree of patient participation in SDM.
(6) The Multidimensional Scale of Perceived Social Support [34]: MSPSS was developed by Zimet, and translated by Kee-Lee Chou [35]. Perceived social support was measured with the MSPSS. The scale consists of 12 items (Cronbach’s α = 0.89) with answers ranging from “Completely disagree” to “Completely agree” (1 to 7). The measure assesses social support received from three subscales (4 items each)—friend subscales (Cronbach’s α = 0.95), family subscales (Cronbach’s α = 0.92), and significant other subscales (Cronbach’s α = 0.91). The general scores were calculated by adding the 12 items together (ranging from 12 to 84). A higher total score indicates higher levels of social support for patients.

#### 2.4.4. Data Analysis

The questionnaire will be checked by two people, and scientific statistical methods will be used for rigorous statistical analysis. The quantitative data collected through interviews will be analyzed using SPSS26.0. Descriptive statistics will be used to present all the variables (Table 1). Mann–Whitney U-tests or multi-sample rank-sum tests will be used to analyze the differences between general information and the level of participation in SDM in patients with SCAD. The variables that are statistically significant after monofactor analysis will be used as independent variables, and participation in SDM by SCAD patients will be used as the dependent variable for conducting multiple logistic regression analyses. The Spearman correlation analysis will be used to describe the correlation between the variables in the TPB model and participation in SDM by SCAD patients. Two-tailed *p*-values will be adopted, and *p* < 0.05 will be considered statistically significant.

### 2.5. Stage 2: Qualitative Study

#### 2.5.1. Sample

To obtain as much information as possible from the interviews, maximum difference sampling will be used. The SCAD patients included in the quantitative study as the target population, and as far as possible, SCAD patients with different levels of SDM participation, with different risk factors for cardiovascular disease, with different ages, with different levels of education, and with different economic statuses representative of the results of the quantitative study will be selected for the interviews. The final sample size will be determined according to the information saturation; that is, the interview will end when no new content and themes emerge, and 10–20 interviewees are expected to be included.

#### 2.5.2. Study Setting and Procedure

(1) The researcher will contact potential interviewees in advance to arrange suitable times and locations for the qualitative interviews. The research will employ face-to-face semi-structured interviews conducted by the same researcher who will undergo comprehensive training in qualitative research methodology. The researcher will engage with the interviewees through a question-and-answer format following the structure of the interview outline. Based on the interviewees’ responses, the order of the outline will be adapted and follow-up questions will be posed to gather as much rich information as possible.
(2) The interview outline of this study is developed based on the theory of planned behavior and relevant literature and will be revised according to the survey results of the quantitative stage. Prior to the formal interviews, two respondents who meet the inclusion criteria will be pre-interviewed. Based on the results of these pre-interviews, the formal interview outline will be revised and finalized, and the interviewing techniques will be improved.
(3) Before the interview, the researcher will prepare the interview outline, informed consent form, writing implement, interview transcript, and notebook, and adjust the audio recording equipment. At the outset of the interview, the researcher will elucidate the purpose and content of the interview, as well as the rationale for recording, and obtain the interviewee’s signature on the informed consent form, indicating their understanding and consent. The location of the interview will be selected with the intention of providing the interviewees with a setting that will be convenient, comfortable, and relaxed. The primary locations selected for the interviews in this study will be quiet wards, conference rooms, and disposal rooms.
(4) During the interview process, the researcher will additionally record the interviewees’ expressions, emotional responses, body movements, and other non-verbal behaviors. After the interview, a diary will be written in a timely manner to document the researcher’s impressions and facilitate data analysis.
(5) According to the principle of data saturation in qualitative research, the interview will be concluded when the researcher cannot extract new themes and content from the interview data.

#### 2.5.3. Instruments

Semi-structured Interview Outline (Table 2).

The interview outline of this study is developed based on the theory of planned behavior and relevant literature, and will be revised according to the survey results of the quantitative stage.

#### 2.5.4. Data Analysis

The qualitative data analysis will be performed using thematic coding methods. The interview recording will be transcribed word for word within 24 h after the end of the interview, and the recording will be repeatedly listened to to ensure the accuracy of the transcribed content, and the interviewees’ modal words and other content will be supplemented with the interview record form. The transcribed text is then imported into the Nvivo12.0 software for management, encoding, and analysis. The transcription and analysis of the data will be carried out independently by two researchers, and consensus will be reached through discussion and the questioning of a third researcher in the case of disagreement.

### 2.6. Integration Study

**Figure 3:**
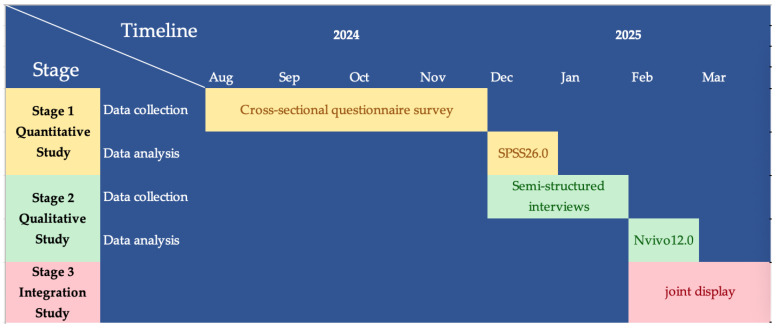
Gantt chart for this mixed method study.

In order to comprehensively analyze the results of the quantitative and qualitative studies, we will integrate the quantitative and qualitative results through a joint display [36] in a mixed methods approach using TPB as a framework. We will combine the current status and factors influencing the participation of SCAD patients in SDM in the quantitative study and the themes, sub-themes, and related results extracted from the qualitative study, and present them together visually so that we can compare the findings of the different methods and analyze them comprehensively. Figure 3 presents the Gantt chart illustrating the timeline for the mixed method study.

### 2.7. Patient and Public Involvement

Patients and the public were not directly engaged in the development process of the protocol.

## 3. Discussion

### 3.1. Strengths

The study employs a mixed method approach with an explanatory sequential design, incorporating both qualitative and quantitative methods, to comprehensively understand the factors that influence SCAD patients’ participation in SDM. The SCAD patients who report low, moderate, and high levels of participation in SDM and have different demographic and clinical characteristics will be purposively selected to be interviewed to understand their different experiences and explore their promoting and hindering factors. Furthermore, these findings can provide a reference for the design of future intervention studies, as well as provide healthcare providers with targeted information and communication to help SCAD patients make the most appropriate decisions.

Cost–benefit analysis (CBA) and expected utility maximization (EUM) are widely recognized as rational criteria [37]. However, TPB provides an additional perspective as a rational criterion. Firstly, TPB emphasizes understanding and predicting human behavior by focusing on intentions, attitudes, subjective norms, and perceived behavioral control [23]. This makes it particularly useful for interventions to change specific behaviors, like health-related practices [23]. This will have significant implications for our subsequent intervention studies. This can be more actionable than the more abstract calculations of CBA and EUM, which might not always translate directly into practical strategies.

Secondly, unlike CBA and EUM, which primarily consider economic outcomes and individual preferences, TPB incorporates social and psychological factors. It acknowledges that people’s behaviors are influenced by their beliefs about what others think they should do (subjective norms) and their perceived ability to perform the behavior (perceived behavioral control) [38].

Finally, in the SDM process, patients’ attitudes toward the different treatment options are crucial. If a patient has a positive attitude toward a particular treatment because they believe it will lead to better health outcomes, they are more likely to consider and choose that option during the decision-making process. SDM allows these attitudes to be explicitly discussed and considered [23]. From the results, the outcome of SDM is a shared decision that ideally reflects a strong intention to follow the agreed-upon treatment plan. Because the decision is made collaboratively and fully considering the patient’s views and perceived control, it is more likely that the patient will follow through with the treatment, which is consistent with the TPB’s emphasis on the link between intention and behavior.

### 3.2. Limitations

Firstly, our study participants will be recruited using convenience sampling from just one clinical setting. The single setting and the sampling methodology are non-probabilistic, limiting the findings’ generalizability. Secondly, for the elderly with lower levels of education, there is a possibility that they may not be able to comprehend the content of the questionnaires and interview outlines, which could potentially lead to communication difficulties. It is incumbent upon the researcher to elucidate the questionnaire and interview outline to the elderly in simple and clear statements. This may require a greater investment of time and energy on the part of the patient, which could potentially impede the research process.

TPB also has some limitations that should be mentioned. Firstly, TPB assumes that individuals make decisions based on the rational assessments of available information. In reality, however, people often make decisions based on past behavior and habits, which are not fully captured by the theory [39]. This may lead to overestimating the predictive power of attitudes, subjective norms, and perceived behavioral control in some situations. Secondly, TPB assumes that individuals have a degree of control over their behavior. However, in some cases, people may have limited control due to factors such as their medical condition, mental health problems, or environmental constraints. When patients lack control over their behavior, TPB’s predictive power is reduced because intentions may not be translated into action [39].

### 3.3. Dissemination

The corresponding results and conclusions will be disseminated in correlative conferences and journals after the completion of the study, and data pertinent to this study’s results will be freely available from the corresponding author via reasonable request. Furthermore, we will disseminate and share the findings with the public and relevant health administration organizations. This may prompt discourse surrounding patient participation in SMD and potentially shape patients’ understanding and attitudes towards SMD, thereby enhancing the actual level of patient involvement in SMD and benefiting patients.

## 4. Conclusions

SDM fosters an equitable partnership whereby both the healthcare provider and the patient collectively assume responsibility for making medical decisions. An explanatory sequential mixed methods design will be used to explore the study aims. The results will provide an in-depth analysis of the factors that influence SCAD patients’ participation in SDM and their participation experiences, revealing their unique clinical challenges and providing valuable insights. These findings aim to strengthen communication between healthcare providers and patients with SCAD, and provide targeted recommendations and references for clinical improvement and the promotion of SCAD patients’ participation in SDM. However, there are some limitations in the design and theory of this study, which can be discussed in future studies.

## Figures and Tables

**Table 1:** Statistical test information table.

Statistical Test Information Table			
Num	Tests	Variables	Type of Information
1	descriptive statistics	demographic and clinical characteristics, participation in SDM, patient attitudes, decision self-efficacy, intention, and social support	Frequency, percentage; Mean ± SD or M (P_25_, P_75_) (normality or not)
2	monofactor analysis	sex, BMI, ethnicity, place of residence, working conditions, medical payment method, stent implantation or not, and the first time to be hospitalized for SCAD or not	Mann–Whitney U-tests
		age, marital status, education level, average annual household income, drinking status, smoking status, duration of illness, severity of angina, and number of diseased coronary arteries	multi-sample rank-sum tests
3	multiple logistic regression analyses	Variables that are statistically significant after monofactor analysis	independent variables
		participation in SDM	dependent variable
4	Spearman correlation analysis	participation in SDM (Single dimension)	
		patient attitudes (Single dimension)	
		decision self-efficacy (Single dimension)	
		intention (Single dimension)	
		social support (friends support, family support, and significant other support)	

Demographic and clinical characteristics: sex, BMI, ethnicity, place of residence, working conditions, medical payment method, stent implantation or not, and the first time to be hospitalized for SCAD or not; age, marital status, education level, average annual household income, drinking status, smoking status, duration of illness, severity of angina, and number of diseased coronary arteries.

**Table 2:** Semi-structured Interview Outline.

Opening remarks	I appreciate your interest in this study. I will ask you some questions about your involvement in SDM during this hospitalization. Your responses will be recorded, but your identity will remain confidential.	
SDM Participatory experience	1. Can you recall when the most impressive/recent medical decision was made during your hospitalization? 2. Please describe in detail what was the decision-making process? (Decision on what? How did you participate in the decision? How was the decision made?) ① What actions did you take before making the decision? ② How did you make the decision when the real decision was made? (Please describe in detail) ③ After making the decision, reviewing the whole process, what do you think about the decision-making process?	
Participation in SDM Impact Factors	Intention	① How would you like to be involved in decision-making during your hospitalization? Why do you wish to participate in decision making in this way? ② What do you think led to your willingness to participate in shared decision-making being consistent/inconsistent with your actual behavior in decision-making participation?
	Attitude	① What is your attitude towards participation in shared decision-making? Why do you think so? ② What do you think are the advantages and disadvantages of participating in shared decision-making for you? What are the effects? (For you? Your children? Your family? Others?) ③ How do you feel about your decision-making participation behavior in this way? (Please describe in detail)
	Subjective Norm	①What influence does your partner/family/friends/healthcare provider/other individuals or groups have on your participation in SDM/taking this decision-making participatory behavior? Why did/didn’t it have an impact?
	Perceived behavioral control	① How confident are you in participating in SDM? What would affect your confidence in participating in SDM? ② What aspects of your confidence/non-confidence/competence do you think have led to your decision-making participation behavior in this way? (Please describe in detail)
	Other factors	① What other factors do you think cause/hinder your participation in sharing decisions? ② What other factors do you think may have caused/hindered you from making other decision-making participation behaviors?
Closing remarks	1. Is there anything you would like to share or add about today’s interview?	

## Data Availability

Any data generated from this study will be available for sharing upon the submission of a request to the primary investigator of this study.

## References

[1] Weiss G.B. (1985). Paternalism Modernised. J. Med. Ethics.

[2] Barry M.J., Edgman-Levitan S. (2012). Shared Decision Making—The Pinnacle of Patient-Centered Care. N. Engl. J. Med..

[3] Joseph-Williams N., Elwyn G., Edwards A. (2024). Twenty-One Years of the International Shared Decision Making Conference: Lessons Learnt and Future Priorities. BMJ Evid. -Based Med..

[4] Stiggelbout A.M., Pieterse A.H., De Haes J.C.J.M. (2015). Shared Decision Making: Concepts, Evidence, and Practice. Patient Educ. Couns..

[5] Dennison Himmelfarb C.R., Beckie T.M., Allen L.A., Commodore-Mensah Y., Davidson P.M., Lin G., Lutz B., Spatz E.S. (2023). Shared Decision-Making and Cardiovascular Health: A Scientific Statement from the American Heart Association. Circulation.

[6] Roth G.A., Abate D., Abate K.H., Abay S.M., Abbafati C., Abbasi N., Abbastabar H., Abd-Allah F., Abdela J., Abdelalim A. (2018). Global, Regional, and National Age-Sex-Specific Mortality for 282 Causes of Death in 195 Countries and Territories, 1980–2017: A Systematic Analysis for the Global Burden of Disease Study 2017. Lancet.

[7] Mensah G.A., Fuster V., Murray C.J.L., Roth G.A., Mensah G.A., Abate Y.H., Abbasian M., Abd-Allah F., Abdollahi A., Abdollahi M. (2023). Global Burden of Cardiovascular Diseases and Risks, 1990–2022. J. Am. Coll. Cardiol..

[8] Hu S.-T., Wang Z.-W. (2023). Overview of China Cardiovascular Health and Disease Report 2022. Chin. Cardiovasc. Res..

[9] Bhatt D.L. (2018). Percutaneous Coronary Intervention in 2018. JAMA.

[10] Bangalore S., Maron D.J., Stone G.W., Hochman J.S. (2020). Routine Revascularization Versus Initial Medical Therapy for Stable Ischemic Heart Disease: A Systematic Review and Meta-Analysis of Randomized Trials. Circulation.

[11] Knops A.M., Goossens A., Ubbink D.T., Legemate D.A., Stalpers L.J., Bossuyt P.M. (2013). Interpreting Patient Decisional Conflict Scores: Behavior and Emotions in Decisions about Treatment. Med. Decis. Mak..

[12] Chhatriwalla A.K., Decker C., Gialde E., Catley D., Goggin K., Jaschke K., Jones P., deBronkart D., Sun T., Spertus J.A. (2019). Developing and Testing a Personalized, Evidence-Based, Shared Decision-Making Tool for Stent Selection in Percutaneous Coronary Intervention Using a Pre-Post Study Design. Circ. Cardiovasc. Qual. Outcomes.

[13] Virani S.S., Newby L.K., Arnold S.V., Bittner V., Brewer L.C., Demeter S.H., Dixon D.L., Fearon W.F., Hess B., Johnson H.M. (2023). 2023 AHA/ACC/ACCP/ASPC/NLA/PCNA Guideline for the Management of Patients With Chronic Coronary Disease: A Report of the American Heart Association/American College of Cardiology Joint Committee on Clinical Practice Guidelines. Circulation.

[14] Lawton J.S., Tamis-Holland J.E., Bangalore S., Bates E.R., Beckie T.M., Bischoff J.M., Bittl J.A., Cohen M.G., DiMaio J.M., Don C.W. (2022). 2021 ACC/AHA/SCAI Guideline for Coronary Artery Revascularization: Executive Summary: A Report of the American College of Cardiology/American Heart Association Joint Committee on Clinical Practice Guidelines. Circulation.

[15] Stacey D., Légaré F., Lewis K., Barry M.J., Bennett C.L., Eden K.B., Holmes-Rovner M., Llewellyn-Thomas H., Lyddiatt A., Thomson R. (2017). Decision Aids for People Facing Health Treatment or Screening Decisions. Cochrane Database Syst. Rev..

[16] Zhao J.-J., Guo B.-M., Liu C.-C., Zhang Y., Zhao L., Zhu J. (2017). Study on the expected benefit of percutaneous coronary intervention and its influencing factors in patients with stable coronary heart disease. Chin. J. Nurs..

[17] Ming J., Wei Y., Ke X., He L.-Y., Li D., Sun H., Xu D., Chen Y.-Y. (2018). Study on patient participation in decision making in clinical application of drug-coated stent technology. Chin. Health Policy Res..

[18] Min J., Yu Y.-W., Lee J., Yeon S., Park H.-N., Lee J.S., Courneya K.S., Park H.S., Kim S.I., Jeon J.Y. (2022). Application of the Theory of Planned Behavior to Understand Physical Activity Intentions and Behavior among Korean Breast Cancer Survivors. Support Care Cancer.

[19] Yang Q. (2024). Understanding the Associations between Adolescents’ Exposure to E-Cigarette Information and Vaping Behavior through the Theory of Planned Behavior. Health Commun..

[20] Haubenstricker J.E., Lee J.W., Segovia-Siapco G., Medina E. (2023). The Theory of Planned Behavior and Dietary Behaviors in Competitive Women Bodybuilders. BMC Public Health.

[21] Carvajal D.N., Mudafort P.C.R., Barnet B., Blank A.E. (2020). Contraceptive Decision Making among Latina Immigrants: Developing Theory-Based Survey Items. Hisp. Health Care Int..

[22] Parker M.G., Hwang S.S., Forbes E.S., Colvin B.N., Brown K.R., Colson E.R. (2020). Use of the Theory of Planned Behavior Framework to Understand Breastfeeding Decision-Making among Mothers of Preterm Infants. Breastfeed Med..

[23] Ajzen I. (1991). The Theory of Planned Behavior. Organ. Behav. Hum. Decis. Process..

[24] Stuart A., Ord K. (1995). Kendall’s Advanced Theory of Statistics. J. Am. Stat. Assoc..

[25] Group of Interventional Cardiology, Society of Cardiology, Chinese Medical Association, Atherosclerosis and Coronary Heart Disease Group, Society of Cardiology, Chinese Medical Association, Thrombus Prevention and Treatment Professional Committee of Cardiovascular Physicians Branch of Chinese Medical Doctor Association (2018). Editorial Board of the Chinese Journal of Cardiovascular Diseases Guidelines for diagnosis and treatment of stable coronary heart disease. Chin. J. Cardiovasc. Dis..

[26] Arnetz J.E., Höglund A.T., Arnetz B.B., Winblad U. (2008). Development and Evaluation of a Questionnaire for Measuring Patient Views of Involvement in Myocardial Infarction Care. Eur. J. Cardiovasc. Nurs..

[27] Shen H. (2000). A Study of Factors Influencing Patient Participation and Impact on Treatment Outcomes. Master’s Thesis.

[28] Bunn H., O’Connor A. (1996). Validation of Client Decision-Making Instruments in the Context of Psychiatry. Can J. Nurs. Res..

[29] Wang S.T., Ye Z.X., Li Y.Y., Liu F., Li L. (2021). Reliability and Validity Testing of the Chinese Version of Decision Self-Efficacy Scale among Patients with Primary Liver Cancer for Treatment Decision-Making. Nurs. J. Chin. People’s Lib. Army.

[30] Ende J., Kazis L., Ash A., Moskowitz M.A. (1989). Measuring Patients’ Desire for Autonomy: Decision Making and Information-Seeking Preferences among Medical Patients. J. Gen. Intern. Med..

[31] Kim M., Smith D.H., Gu Y. (1999). Medical Decision Making and Chinese Patients’ Self-Construals. Health Commun..

[32] Kriston L., Scholl I., Hölzel L., Simon D., Loh A., Härter M. (2010). The 9-item Shared Decision Making Questionnaire (SDM-Q-9). Development and psychometric properties in a primary care sample. Patient Educ. Couns..

[33] Luo B.H., Xiao S.Y. (2019). Reliability and validity of the patient version of the Chinese version of the Doctor-Patient Co-decision Questionnaire. J. Cent. South Univ. (Med. Sci.).

[34] Zimet G.D., Dahlem N.W., Zimet S.G., Farley G.K. (1988). The Multidimensional Scale of Perceived Social Support. J. Personal. Assess..

[35] Chou K.L. (2000). Assessing chinese adolescents’ social support: The multidimensional scale of perceived social support. Personal. Individ. Differ..

[36] Guetterman T.C., Fetters M.D., Creswell J.W. (2015). Integrating Quantitative and Qualitative Results in Health Science Mixed Methods Research Through Joint Displays. Ann. Fam. Med..

[37] Barnes B.A. (1982). Cost-Benefit and Cost-Effective Analysis in Surgery. Surg. Clin. N. Am..

[38] Ajzen I. (2020). The Theory of Planned Behavior: Frequently Asked Questions. Hum. Behav. Emerg. Technol..

[39] Ajzen I. (2011). The Theory of Planned Behaviour: Reactions and Reflections. Psychol. Health.

